# Joint EANM, SNMMI and IAEA enabling guide: how to set up a theranostics centre

**DOI:** 10.1007/s00259-022-05785-x

**Published:** 2022-04-11

**Authors:** Ken Herrmann, Luca Giovanella, Andrea Santos, Jonathan Gear, Pinar Ozgen Kiratli, Jens Kurth, Ana M. Denis-Bacelar, Roland Hustinx, Marianne Patt, Richard L. Wahl, Diana Paez, Francesco Giammarile, Hossein Jadvar, Neeta Pandit-Taskar, Munir Ghesani, Jolanta Kunikowska

**Affiliations:** 1 Department of Nuclear Medicine, University of Duisburg-Essen, Duisburg, Germany; 2 German Cancer Consortium (DKTK)-University Hospital Essen, Essen, Germany; 3 Clinic for Nuclear Medicine and Molecular Imaging, Imaging Institute of Southern Switzerland, Ente Ospedaliero Cantonale, Bellinzona, Switzerland; 4 Department of Nuclear Medicine, Hospital Cuf Descobertas, Lisbon, Portugal; 5 Joint Department of Physics, Royal Marsden NHS Foundation Trust, Sutton, UK; 6 Department of Nuclear Medicine, Hacettepe University, Ankara, Turkey; 7 Department of Nuclear Medicine, Rostock University Medical Center, Rostock, Germany; 8 National Physical Laboratory, Teddington, UK; 9 Division of Nuclear Medicine and Oncological Imaging, University Hospital of Liège, Liège, Belgium; 10 GIGA-CRC in vivo imaging, University of Liège, Liège, Belgium; 11 Department for Nuclear Medicine, University Hospital Leipzig, Leipzig, Germany; 12 Department of Radiology, Washington University School of Medicine, St. Louis, MO USA; 13 Nuclear Medicine and Diagnostic Imaging Section, Division of Human Health, Department of Nuclear Sciences and Application, International Atomic Energy Agency, Vienna, Austria; 14 Division of Nuclear Medicine, Department of Radiology, University of Southern California, Los Angeles, CA USA; 15 Department of Radiology, Memorial Sloan Kettering Cancer Center, New York, NY USA; 16 Diagnostic, Molecular & Interventional Radiology, Icahn School of Medicine at Mount Sinai, New York, NY USA; 17 Nuclear Medicine Department, Medical University of Warsaw, Warsaw, Poland

**Keywords:** Theranostics, Radionuclide theranostics, Nuclear medicine, PSMA, PRRT

## Abstract

The theranostics concept using the same target for both imaging and therapy dates back to the middle of the last century, when radioactive iodine was first used to treat thyroid diseases. Since then, radioiodine has become broadly established clinically for diagnostic imaging and therapy of benign and malignant thyroid disease, worldwide. However, only since the approval of SSTR2-targeting theranostics following the NETTER-1 trial in neuroendocrine tumours and the positive outcome of the VISION trial has theranostics gained substantial attention beyond nuclear medicine. The roll-out of radioligand therapy for treating a high-incidence tumour such as prostate cancer requires the expansion of existing and the establishment of new theranostics centres. Despite wide global variation in the regulatory, financial and medical landscapes, this guide attempts to provide valuable information to enable interested stakeholders to safely initiate and operate theranostics centres. This enabling guide does not intend to answer all possible questions, but rather to serve as an overarching framework for multiple, more detailed future initiatives. It recognizes that there are regional differences in the specifics of regulation of radiation safety, but common elements of best practice valid globally.

## Introduction

The theranostics concept—i.e. using the same target for both imaging and therapy—has been the cornerstone of therapeutic nuclear medicine since the introduction for treatment of thyroid disease in the early 1940s. Despite the fact that iodine-131 (^131^I)- and yttrium-90 (^90^Y)-radiolabelled anti-CD20 antibodies showed excellent long-term clinical outcomes in low-grade non-Hodgkin lymphomas [1–3], these agents have largely been replaced by non-radioactive therapies, mainly due to market forces and the relative ease of delivering non-radioactive treatments. The success story of iodine theranostics in thyroid diseases and the recent approval of lutetium-177 [^177^Lu]Lu-DOTATATE following the landmark NETTER-1 [1] trial have increased the applications of targeted radionuclide therapies. The expansion of the theranostics concept beyond thyroid cancer and neuroendocrine tumours towards higher incidence diseases like prostate cancer (and subsequently to other tumours) shifts nuclear medicine and radionuclide therapy into the spotlight of modern cancer therapies. VISION, a prospective randomised phase 3 trial, showed that in prostate cancer, the most common and second most fatal cancer in men, the use of up to six cycles of [^177^Lu]Lu-PSMA-617 increased the median overall survival by 4 months (15.3 vs. 11.3 months, HR 0.62; 95% CI 0.52 − 0.74; *P* < 0.001) [2] in patients with metastatic castration-resistant prostate cancer (mCRPC).

A tremendous increase in the demand for theranostics procedures can be expected in anticipation of FDA and EMA approval of [^177^Lu]Lu-PSMA-617, and this projected surge in demand for both theranostics infrastructure and appropriately skilled professional staff will pose a challenge and opportunity for healthcare systems. Even in countries with a strong track record in radionuclide theranostics, the existing infrastructure may be insufficient to meet the growing demand [3, 4]. Thus, theranostics and radionuclide therapy need to get ready for the demand from cancer patients, referring physicians and society. Here, we provide an enabling guide for p stakeholders interested in setting up a dedicated theranostics centre. Special attention is given to regulatory considerations and requirements, logistical and technical challenges, medical considerations including training, collaboration with clinical partners and treatment indications and important lessons learnt from early adopters of theranostics. We also provide advice for troubleshooting during creating a theranostics service. This guide does not cover the specific requirements associated with the in-house production of radiopharmaceuticals since there is no global harmonisation, and national laws differ considerably.

## Regulatory, logistical and technical considerations

The design, construction and subsequent operation of a theranostics service have to be guided by the fundamentals of radiation protection established by the appropriate regulatory agencies. In Europe, the International Atomic Energy Agency (IAEA) through the International Basic Safety Standards (BSS)[5] are a set of consensus requirements derived from knowledge of radiation biology and radiation protection, respectively [6]. In the USA, the Nuclear Regulatory Commission (NRC) governs safety standards and delegates’ responsibilities to specific states (Agreement States) in many instances. The European Commission Directive 2013/59/EURATOM is a legal act that establishes the recommendations and requirements of the BSS and ICRP for EU countries, which have been transposed into national law by the Member States. Sections 2 and 3 of the BSS specify that requirements that apply to all existing and planned exposure situations must be considered when establishing and operating a theranostics centre.

The BSS requires that legal entities apply to the regulatory authority for a license. Therefore, the regulatory basis for operating a theranostics centre is a radioactive material license (RAM), in accordance with the national regulations and laws governing the handling of radioactive materials for medical applications, as defined in ICRP Publication 105 [7]. This must cover all aspects of both diagnostic and therapeutic use of radiopharmaceuticals. Prerequisites for applying for a RAM license include the existence of adequate infrastructure, sufficient personnel (including trained physicians, technologists, nursing staff, a radiation safety officer (RSO), a medical physics expert (MPE)), sufficient means of radiation protection and processes for discharge management of treated patients and handling of radioactive waste and sewage. To this end, several requirements must be met, depending on the respective spectrum of diagnostics and therapies applied and the radiopharmaceuticals used. In the USA, regulations differ but require a suitable radiation license and appropriately qualified authorized users to allow administration of the radiopharmaceutical therapies.

## Radionuclides and radiopharmaceuticals used

A commonly used theranostics pair is gallium-68 (^68^Ga) for PET/CT diagnostics and ^177^Lu for therapy. In the USA, [^64^Cu]Cu-DOTA-TATE is commonly used in addition to [^68^Ga]Ga- DOTA-TATE or DOTA-TOC. However, ^90^Y is also occasionally therapeutic, as are fluorine-18 (^18^F)- and technetium-99 m (^99m^Tc)-labelled diagnostic compounds. Table 1 summarises the main properties of these radionuclides.

**Table 1:** Physical characteristics of the commonly used theranostics pairs ^68^ Ga/^177^Lu and ^68^ Ga/^90^Y

Radionuclides	Physical characteristics*			Pharmaceuticals^†^	Use	
	Energy [keV]		Half-life			
	Gamma	Beta or Alpha				
^68^Ga	511 (caused by annihilation)	1899 ($\beta$^+^)	1.13 h	[^68^Ga]Ga-PSMA-11 [^68^Ga]Ga-PSMA-I&T [^68^Ga]Ga-DOTA-TATE (NETSPOT ™) [^68^Ga]Ga-DOTA-TOC (SomaKIT TOC ®)		Diagnostic
^18^F	511 (caused by annihilation)	634 ($\beta$^+^)	1.83 h	Piflufolastat F18 (Pylarify®) [^18^F]F PSMA-1007 [^18^F]DCFPyL		
^177^Lu	113 (6%) 208 (11%)	498 ($\beta$^−^)	6.73 d	[^177^Lu]Lu-PSMA-617 [^177^Lu]Lu-PSMA-I&T [^177^Lu]Lu-DOTA-TATE (Lutathera®)		Therapy
^90^Y	Bremsstrahlung	2280 ($\beta$^−^)	2.67 d	[^90^Y]Y-DOTA-TOC		
^99m^Tc	140 (89%)	not relevant	6.01 h	[^99m^Tc]Tc-MDP [^99m^Tc]Tc-DPD [^99m^Tc]Tc-HDP		Diagnostic
^223^Ra	154 (6%) 269 (14%)	5716 ($\alpha$), 5606 ($\alpha$), 6819 ($\alpha$), 7386 ($\alpha$), 6623 ($\alpha$) 1370 ($\beta$^−^) 1420 ($\beta$^−^)	11.44 d	^223^RaCl_2_ (Xofigo ®)		Therapy

^*^Data are extracted from The Lund/LBNL Nuclear Data Search V 2.0 (http://nucleardata.nuclear.lu.se/toi/)

^†^Without claim to completeness

With ^177^Lu, attention must be paid to the underlying manufacturing pathway, which may result in unwanted long-lived accompanying nuclides that require special consideration in terms of storage and disposal of waste depending on local regulations. ^177^Lu is made either by direct neutron irradiation of ^176^Lu targets (^176^Lu (n,γ) reaction) or indirectly as a decay product of the neutron irradiation of ytterbium-176 (^176^Yb (n,γ) reaction), which produces ^177^Yb that decays to ^177^Lu. In the indirect reaction, no long-lived contaminants are created. However, in the direct reaction, small quantities of metastable lutetium-177 (^177m^Lu) with a half-life of 161 days may be present [7]. In this case, ^177m^Lu may account for approximately 0.02% of the total amount of ^177^Lu in the final radiopharmaceutical. ^68^ Ga may either be obtained from a radionuclide generator (^68^Ge/^68^ Ga-generator) or produced by proton irradiation of zinc-68 (^68^Zn(p,n)^68^ Ga). The different production pathways of the radionuclides are associated with different radionuclidic impurities that must be taken into account, i.e. germanium-68 and gallium-67, respectively [8]. The ^90^Y currently available for radiolabelling is of high radionuclidic purity with no relevant amounts of accompanying nuclides [9]. Long-lived radioactive contaminants may require specific regulatory attention.

## Radiation protection and shielding

Shielding of syringes and vials, as well as in some jurisdictions, waste and storage containers, is an important aspect of reducing external exposure among staff, the public and patients. After administration of the radiopharmaceutical, it may be necessary (mainly in Europe, and in some cases in the USA depending on exposure rates) to isolate the patient from other persons, either within the hospital or in the public domain. The type of radiation emitted from the theranostics compound will dictate the extent of shielding required. This can vary from PMMA (polymethyl metacrylate) storage boxes for vials and waste containers, lead pots and tungsten syringe shields, to concrete waste bunkers or lead-lined treatment rooms. This infrastructure must be prepared according to local regulation and must be in place before any activity involving radiation is carried out. Appropriateness of the control measures must also be demonstrated, usually in the form of a written radiation risk assessment that considers radiation protection of both employees and patients. Established risk analysis methods such as failure mode and effects analysis (FMEA) or fault tree analysis (FTA) should be used for this purpose. Compliance with NRC and/or state radiation safety regulations is required in the US.

## Storage of radiopharmaceuticals

Radiopharmaceuticals must be stored in a safe, secure and environmentally appropriate (such as refrigerated or frozen) place to which only the licensee and appropriate staff may have access. In addition, provisions for the safe storage and custody of radioactive materials must be in place, including protection against theft, fire and chemicals. Transport and movement of radioactive materials to, from and within the hospital must be carefully documented so that any radioactive material can be tracked from source to final use and disposal.

## Administration of radiopharmaceuticals

Accurate quantification of the radioactivity administered to the patient is the first step of the radiopharmaceutical administration and traceability chain. A radionuclide calibrator measures the activity and cross-calibrates other equipment. It is therefore essential to ensure that calibration is traceable to primary standards when these are available [10–12].

A well-documented programme for quality assurance (QA) and quality control (QC) is essential to ensure the dependable performance of safe, accurate and reproducible equipment operation and the appropriate clinical administration of radiopharmaceuticals [10, 13, 14]. Following installation of any new instrument, acceptance testing must confirm that the system meets the performance specifications and to provide a baseline for comparison during routine QC. The type and frequency of QC tests should follow national guidelines.

The theranostics compounds can be administered in several ways:

Adequate shielding must be ascertained to avoid undesirable beta and gamma irradiation and to minimise the risk of contamination, e.g. by using hybrid shielding consisting of layers of PMMA and lead/tungsten, which results in attenuation of both beta and gamma radiations and minimizes the occurrence of bremsstrahlung. A syringe is prepared with the therapeutic agent, and the qualified operator administers the drug via correctly placed and patent intravenous access. This is followed by flushing with saline. This method is particularly used for drugs such as PSMA ligands, which do not require specific administration as a bolus. Alternatively, the syringe content can be administered via perfusor or injection pump. To minimize staff radiation exposure it is recommended to use automatic dispensing and semior fully automated infusion pumps for the administration of the radiopharmaceuticals.

## Radioactive waste

Storage for decay is essential for the clearance of radioactive waste containing short-lived radioisotopes, with a half-life of less than 100 days. “Clearance is the removal of radioactive material from regulatory control provided that the radionuclide concentrations are below specific clearance levels” [12].

Waste may be stored for decay and subsequent discharge in a locked, ventilated and properly demarcated room. It is recommended to segregate radionuclides according to the expected time required for their decay (e.g. initial activity and physical half-life). For example, the shorter lived waste from PET/CT diagnostics (syringes, swabs, vials, etc.) should be separated from that of the longer lived radionuclides used for the therapy. There should also be sufficient space in these rooms for interim storage of potentially contaminated items (e.g. patient clothing, patient diapers, perfusors, etc.). The origin of the waste should be recorded to ensure proper identification.

Disposal of aqueous radioactive wastes must strictly follow the recommendations set out in the national regulations. These may allow a limited amount of highly diluted wastewater to be disposed of into the public sewage system or require specific processing such as filtration and/or specific storage systems before release. Local regulatory authorities may also additionally require the facility to regularly assess the environmental and radiological impact of radiation work being undertaken.

If long-lived contaminants such as ^177m^Lu (t1/2 = 160 d) are present in the radiopharmaceutical, the waste (e.g. vials, cannula, infusion lines, swabs, etc.) should be stored separately from other waste until the time limit for disposal according to national law is reached. Specific attention must be paid to isolation, storage and disposal of biohazardous and radioactive waste which may contain patient fluids. Potential contamination of liquid waste (i.e. excreta) with ^177m^Lu must also be considered, and any wastewater treatment or storage facilities used—if applicable—must be inspected for capacity and compliance with regulatory limits. Installation of separate toilets for patients treated with theranostics compounds potentially containing ^177m^Lu is also an option.

## Release of patients after treatment

Prior to injection of a radioactive substance, radiation safety guidance should be given to the patient and family (where applicable) regarding rules of conduct to reduce the potential radiation exposure to others. Release of patients after diagnostic procedures does not require extensive or any (USA) measures, since the physical and effective half-life of radiotracers involved is usually only a few hours. With ^68^Ga-, ^64^Cu-, or ^18^F-based tracers, it is therefore usually sufficient to restrict direct contact between the patient and vulnerable individuals (pregnant women, children) during the hours immediately after patient release. In the USA, no or limited radiation-specific discharge instructions are given following diagnostic procedures. The situation is somewhat different for patient discharged after therapeutic administrations, as the activity levels here are significantly higher. ICRP Publication 94 [15, 16] and IAEA Safety Report No.63 [17] comment on the release of patients after radionuclide therapy. A dose limit of 1 mSv/y for the public and a dose constraint of 5 mSv/episode for caregivers (a family member or paid helper who regularly looks after a child or a sick, elderly, or disabled person) have been proposed as acceptable limits. However, in many countries, there are different limits and specifications that must be followed after therapeutic administration of radionuclides. In [^177^Lu]Lu-PSMA-617 therapy, for instance, patients are typically treated with an activity of 7.4 GBq and the initial dose rate from the patient after the injection is in the order of 50 µSv/h at a distance of 1 m. If, as in many countries, a dose rate threshold of 30 μSv/h at a distance of 1 m is used as release criterion, the therapy can be applied as an outpatient treatment and patients can return home within 6 h of administration [18]. Consideration must be taken when more than one radionuclide therapy per year is administered. For example, if a patient is treated with six cycles of PSMA-targeted therapy per year, the cumulative exposure received by the family members, the caregivers and the public must be taken into account. In this case, exposure to members of the public that the patient has frequent contact with (such as family members, children or co-workers) should be kept below one-sixth of the annual limit after each cycle. The same considerations can be applied to [^177^Lu]Lu-DOTATATE therapies.

For both therapies, the high excretion rate in the first hours after therapy administration must be considered: after 4 h, approximately 50% of the activity may be renally excreted [19, 20]. To be compliant with the dose limits, a system needs to be established to measure or estimate activity in patients before discharge and calculate the exposure that members of the household and public may receive (European standards do not apply in other parts of the world). The result should be recorded. One method of estimating the acceptable activity of radiopharmaceuticals in patients upon discharge from hospital is to calculate the time integral of the ambient equivalent dose rate and compare it with the dose limits. Direct measurement of patient activity before discharge is commonly performed and can be used as a patient-specific guide to minimize radiation exposure to caregivers and the general public. The patient should be given written instructions on precautions for the first few days after discharge. In particular, contact with pregnant women and small children should be avoided. Special attention should be given to the risk of contamination via urine, especially in the case of incontinent patients and children. In some cases, it may be appropriate to mandate hospital isolation due to this risk, even if the external dose rate is deemed adequately low. In some countries, including Germany, Austria and Italy, hospitalisation is in any case mandatory following radionuclide therapy.

## Handling of deceased persons

Despite careful patient selection, death of patients, while receiving therapy or soon after, could happen. Such cases could increase as the use of radiopharmaceutical treatments becomes more widely used. If such situations arise, appropriate measures must be taken to handle the corpse. This includes restricted access to the room occupied by the deceased until a proper decontamination and survey have been completed. Radioactive corpses must be identified as a potential hazard using proper identifiers. In case of leakage of radioactive substances, a body bag is needed. In addition, surveillance may be needed in all stages of disposal [17]. None of this however is currently required in the USA, but careful discussions are commonly held with funeral homes regarding safe handling of patients who have died soon after receiving a radiopharmaceutical therapy.

Handling (preparation for burial or cremation) of a body containing significant radioactivity must be carried out under the supervision of a radiation protection officer [17]. Depending on the national regulations, cremation may be postponed for several days or even weeks. Autopsy is not advisable in such cases and must be kept to a minimum. In consultation with the radiation protection officer, all necessary radiation protection and decontamination measures must be undertaken for personnel, instruments and the workplace.

## Treatment planning, optimisation and verification

Council Directive 2013/59/Euratom calls for the planning, optimisation and verification of all radiotherapy exposures in the geographical areas of the EU. The EANM recently provided guidance on how to interpret the Directive’s statements for NM treatments [21]. Theranostics procedures are the epitome of such exposures, allowing the appropriateness of therapy to be determined via companion diagnostic imaging, followed by post-administration therapy imaging for treatment verification, followed by further diagnostic response imaging. ^68^Ga- is generally accepted as the favoured diagnostic companion for ^177^Lu-based therapies although copper-64 (^64^Cu) is seeing increased application in some settings. Most countries in the EU, North America and the Far East show a fairly high density of PET centres, i.e. at least 1/million [22]. The short half-life of ^68^Ga can make transport to centres difficult unless the production site is a short distance away or production is carried out within an in-house radiopharmacy facility. With the emerging availability of licensed kits for ^68^Ga-labelled tracers, the clinical availability of these compounds and the longer lived tracers such as ^64^Cu will increase as well. Considering the many advantages of PET/CT imaging as a companion diagnostic tool, all efforts should be made to equip the countries still lacking so that equal access to therapies can be achieved, as highlighted by the Lancet Oncology Commission on Medical Imaging and Nuclear Medicine [22]. Scaling up access of imaging, treatment and care quality will produce substantial health and economic benefits, and avert millions of death, but will require initial investment before a return is observed [22].

The complexity of the task and the resources required to implement theranostics will vary depending on the respective radiopharmaceutical, application and desired clinical end-point [23]. Commercial software applications are now available, some of which have FDA/EMA approval and are intended to perform dosimetric evaluation [24]. However, in many centres, software developed in-house is still widely used and remains a valuable option for research purposes and post-therapy dosimetry.

Dosimetry calculations require measurements of the distribution of activity in the targets of interest at different time-points to determine the time-integrated activity [25, 26]. Methods requiring less resourcing include whole-body, blood and bone marrow dosimetry, which use external probe measurements of the activity in a tissue biopsy, blood sample or whole body [27]. The number and frequency of activity measurements require careful consideration and should match the desired biological and clinical endpoint. For wider implementation, there is growing interest in minimising the number of imaging sessions whilst having a sufficient level of accuracy to achieve the desired treatment outcome and reduce patient burden and hospital costs. Nearly all radiopharmaceutical therapies approved in the USA do not require dosimetry as a part of the product labelling.

Publications on general principles and practice of PET/CT imaging as well as information about the EARL accreditation programme for the harmonisation of ^18^F, ^68^Ga and ^89^Zr imaging are provided by the EANM [28, 29]. The EANM guidelines provide recommendations on setting up quantitative SPECT/CT imaging with examples of potential clinical applications and include details on scanner calibration, image acquisition parameters and reconstruction and correction methods [30, 31]. The EANM also provides general guidance on documenting and reporting dosimetry data to facilitate the reproducibility of results [32], as well as a detailed methodology on the evaluation and calculation of uncertainties in absorbed dose calculations [33]. Guidance on logistical and technical considerations when developing quantitative imaging and dosimetry protocols is available for ^131^I[34–38], ^177^Lu[39–41], ^90^Y[42], ^223^Ra[43–45]. The Radiological Society of North America QIBA profiles can inform PET and SPECT applications as can use of the SNMMI Clinical Trials Network Phantoms.

## Medical considerations and reflections

Application of radionuclide therapies requires the involvement and coordination of multiple stakeholders—inter- but also intra-professionally. Whereas in regular patient care the treating physician is also the referring physician, patients undergoing radionuclide therapy are typically followed by clinicians who are not nuclear medicine physicians. In the case of prostate cancer patients, the majority are seen and followed by urologists and medical oncologists. However, radioligand therapies are delivered by authorised users, most typically within nuclear medicine departments. Accordingly, coordination and communication with the treating physician are of utmost importance, especially as the indication of radioligand therapies must be appropriately sequenced in the disease journey of a patient. An active presence and participation of nuclear medicine specialists in the multidisciplinary team is mandatory to ensure acceptance and awareness of radioligand therapies. While in the past our contribution to multidisciplinary teams was often limited to presenting diagnostic images, we now must play a more active role in providing our expertise for potential treatment. Overall, a proactive approach promoting theranostics methods will facilitate the adoption and acceptance of our field by our clinical colleagues. This role change also needs to be reflected in the training of junior doctors and the continued education of board-certified nuclear medicine specialists.

## Integrated care

The success of a theranostics centre highly depends on the level of integration within an oncological practice. Indeed both diagnostic imaging and radioligand therapy have to be embedded within the oncologic workflows to facilitate access to the patient flow controlling clinicians. Not surprisingly the currently most successful theranostics centres are embedded in strong cancer centres focusing on neuroendocrine tumours and prostate cancer. Accordingly in anticipation of an ever-growing number of theranostics indications, a close collaboration with all clinical domains managing cancer patients is very important.

## Intra-professional stakeholders

Apart from the inter-professional complexity of theranostics, it is also important to address the multiple specialties and skill sets involved in the successful operation of a theranostics centre. In addition to medical expertise including both physicians and well-trained support staff such as nurses, the administration of either commercially or locally produced theranostics agents requires the involvement of medical physicists, radiochemists/radiopharmacists and radiation safety experts. Whereas many of the skill sets required for theranostics resemble those needed for diagnostic nuclear medicine procedures, the higher activity levels needed for therapy, the different radionuclides involved and the multiple steps in the process, from validating the indication to delivering the radiopharmaceutical, often calls for a significantly higher degree of knowledge but also requires more time. The less infrastructure and local expertise that is already in place, the more demanding the transition to a state-of-the-art theranostics centre will be. Needless to say, the adjustments required from a centre with experience in delivering high-activity radioiodine therapies will be less onerous than those for a site currently only dealing with diagnostic outpatient procedures or ^223^Ra outpatient treatments.

## Training and education

The expected surge in demand for theranostics centres entails numerous challenges. Accessibility and availability of a skilled, well-trained workforce represent one of the greatest unmet needs, alongside upscaling of the healthcare system to accommodate the expected demand for radionuclide therapies. Training and education of existing board-certified nuclear medicine specialists is of high importance, as is the incorporation of radionuclide therapy and the concept of theranostics into the curricula of the ongoing training programmes for junior doctors. Besides learning how to apply radionuclide therapy, understand the right timing for theranostics and the alternative treatments that could be available, deal with typical toxicity profiles and manage the corresponding side effects, there is also an overall shift towards being more actively involved in patient treatment. While cross-training in radiology is helpful for the diagnostic nuclear medicine procedures, the spectrum of radionuclide therapies rather demands a profound expertise in internal medicine, oncology and/or urology. Experienced theranostics centres in countries such as the Netherlands, Switzerland, Germany, the UK and others should accommodate interested nuclear medicine specialists from elsewhere to acquaint them with the application of radionuclide therapies. In the USA, radiation oncologists can become authorized users, but current training for such practitioners in radiopharmaceutical therapy is often quite limited as their focus is external beam therapy in most centres. Practical training of nuclear medicine technologists is also needed, along with continuous education programmes for the development of skills and dissemination of best practice principles. The success of theranostics and the independence of nuclear medicine are directly dependent upon our success in meeting patient demand. In parallel, the introduction of theranostics fellowships mutually accepted by national and international nuclear medicine and clinical societies needs to be pursued. Nuclear medicine physicians who have attained expertise in theranostics will in turn be able to inform the clinicians about all specific aspects of the new treatments with radionuclides. The goal is for medical oncologists and urologists to reach a similar level of comfort with referring patients for these treatments as that reached by clinicians dealing with clinical indications of ^131^I in care of thyroid diseases. In the USA, the SNMMI has endorsed the usage of the term “nuclear oncologist” to refer to nuclear medicine physicians with special expertise in treating cancer patients with targeted radiopharmaceuticals. This term associated with appropriate training and experience may better reflect the critical role of the oncology focused nuclear medicine physician and warrants further use.

## Lessons learnt

Based on our experience, the most important aspect in preparation for the likely surge in theranostics treatments is to seek advice and experience from centres already actively involved in such treatments. Of the lessons learnt, by far the most important is that careful preparation and planning are key to successful implementation.

With an increase in the breadth of theranostics services being delivered comes a larger variety of patients with a wider range of comorbidities and potential complications in safely delivering a radionuclide therapy. Historically, with I-131 thyroid treatments we have been privileged in treating relatively young and healthy patients. Whilst Xofigo® ([^223^Ra]RaCl_2_) treatments brought in an older, frailer population, the reduced radiation risks from the alpha emitter meant that therapies could still be safely delivered in high numbers as an outpatient service in most countries.

PSMA ligand treatments, particularly when radiolabelled with Lu-177, do not necessarily benefit from the same logistical advantages, and thought should be given to the potential complications that could arise from treating such patients. Most notable, from experience, is the increase in the number of patients presenting with some form of lower urinary tract symptom. The degree of incontinence will vary from patient to patient and may be controllable through the wearing of absorbent diapers, or through external, self-inserted or semi-permanent catheterisation. Artificial sphincters and other interventions have also been observed. For a treatment where the primary form of excretion is via the urinary system, this aspect should not be overlooked. A thorough and clear patient history is required so that control measures can be put in place to deal with these complications and there are no surprises on the day of therapy and once the patient returns home.

Consideration should also be given to the patient after treatment. Responsibility for the radiation and potential risks that may occur to the patient, the public and the environment do not stop after the patient has left the hospital. Contingency planning is required to deal with the unexpected, be it disease, treatment, or unrelated emergency care. Examples that have been experienced include blood transfusions for anaemia, orthopaedic surgical interventions and even patient death [46]. It should be recognised that theranostics treatments will impact surrounding and local hospitals in addition to those delivering the radionuclide therapy. It is also likely that the receiving centre and staff will not have the expert knowledge or facilities to deal with radioactive patients or potentially will not possess the required licenses to administer radioactivity or to handle such a patient on site. Good communication and coordination between centres are therefore paramount.

With the expected demand for treatments, outpatient or day case administrations are appealing with a view to increasing patient throughput. However, patient preparation and treatment delivery should not be rushed. Even in centres and countries where treatments can be delivered as a day case, preparations should be in place to respond to delays and contingency plans should be in place to admit the patient overnight, should the need arise. Until the number of theranostics centres increases, extended patient travel time can be expected as current centres cover a wider geographical area. Radiation restrictions during this period should be considered and guidance given as to whether it is more appropriate for the patient to stay in local accommodation rather than undertake a lengthy journey home immediately after therapy.

## Providing points of contact

Promoting theranostics and the scale-up of sites providing access to radionuclide therapy is a joint effort involving multiple professional societies such as EANM and SNMMI, international agencies such as IAEA, but also multiple industry-driven initiatives. A very solid source of information are procedural guidelines promoting the use of innovative diagnostic and therapeutic radionuclides such as [^68^ Ga]Ga-PSMA ligands [47], [^177^Lu]Lu-PSMA ligands [48], [^223^Ra]RaCl_2_ [49], and on a more general level peptide receptor radionuclide therapy [31], among many others. The leading professional societies generally attempt to provide early guidance on how to use novel theranostics, even in cases where clinical evidence is still lacking. For a detailed review, both EANM and SNMMI provide direct access to an overview of procedural guidelines [46, 50]. In addition, several EANM committees, such as the EANM Oncology & Theranostics Committee, or the SNMMI Therapy Centre of Excellence serve as an entry point for individuals requesting assistance or information on how to promote theranostics at local level. More recently, multiple joint initiatives have been launched, paving the way for an understanding of theranostics within the oncological community and facilitating the increased exchange between clinicians and nuclear medicine experts. A pioneering example of this is the joint ESMO/EANM initiative offering advanced courses on diagnostic and therapeutic applications of nuclear medicine in oncology. Additional industry-driven initiatives have recently been announced and will also provide very valuable sources of education and training.

## Summary

The expansion of theranostics applications beyond thyroid cancer and neuroendocrine tumours to a higher-incidence disease such as prostate cancer is triggering the up-scaling of existing and new theranostics centres. This guide establishes an overarching framework helping practitioners to understand what is needed and required to set up a theranostics centre. Despite a widely varying regulatory, financial and medical landscape, the nuclear medicine community will doubtlessly prove capable of responding to the expanding practice in this field. The era of theranostics offers a great opportunity to improve patient care, and theranostics will become a mainstay of personalized cancer treatment. As a community we have the experience and facilities to deliver, with careful preparation and collaboration we will see expansion, and will be ready and able to respond to the demand placed upon us as theranostics continues to develop.

## Declarations

### Liability

This guideline summarizes the views of the co-authoring EANM Committee members, SNMMI representatives and IAEA co-authors. It reflects recommendations for which the EANM, the SNMMI and the IAEA cannot be held responsible. The recommendations should be taken into the context of good practice of nuclear medicine and do not substitute for national and international legal or regulatory provisions.
